# Root-Cause Analysis of Persistently High Maternal Mortality in a Rural District of Indonesia: Role of Clinical Care Quality and Health Services Organizational Factors

**DOI:** 10.1155/2018/3673265

**Published:** 2018-02-22

**Authors:** Mohammad Afzal Mahmood, Ismi Mufidah, Steven Scroggs, Amna Rehana Siddiqui, Hafsa Raheel, Koentijo Wibdarminto, Bernardus Dirgantoro, Jorien Vercruyssen, Hayfaa A. Wahabi

**Affiliations:** ^1^School of Public Health, University of Adelaide, Adelaide, SA, Australia; ^2^Kutai Kartanegara District Department of Health, East Kalimantan, Indonesia; ^3^Women's and Children's Hospital, Adelaide, SA, Australia; ^4^Aga Khan University, Karachi, Pakistan; ^5^King Saud University, Riyadh, Saudi Arabia; ^6^Parikesit Hospital, Kutai Kartanegara, East Kalimantan, Indonesia; ^7^Flinders Medical Centre, Bedford Park, SA, Australia; ^8^Chair of Evidence-Based Healthcare and Knowledge Translation, College of Medicine, King Saud University, Riyadh, Saudi Arabia

## Abstract

**Background:**

Despite significant reduction in maternal mortality, there are still many regions in the world that suffer from high mortality. District Kutai Kartanegara, Indonesia, is one such region where consistently high maternal mortality was observed despite high rate of delivery by skilled birth attendants.

**Method:**

Thirty maternal deaths were reviewed using verbal autopsy interviews, terminal event reporting, medical records' review, and Death Audit Committee reports, using a comprehensive root-cause analysis framework including Risk Identification, Signal Services, Emergency Obstetrics Care Evaluation, Quality, and 3 Delays.

**Findings:**

The root causes were found in poor quality of care, which caused hospital to be unprepared to manage deteriorating patients. In hospital, poor implementation of standard operating procedures was rooted in inadequate skills, lack of forward planning, ineffective communication, and unavailability of essential services. In primary care, root causes included inadequate risk management, referrals to facilities where needed services are not available, and lack of coordination between primary healthcare and hospitals.

**Conclusion:**

There is an urgent need for a shift in focus to quality of care through knowledge, skills, and support for consistent application of protocols, making essential services available, effective risk assessment and management, and facilitating timely referrals to facilities that are adequately equipped.

## 1. Introduction

Maternal healthcare has received significant emphasis in the last two decades globally, and system-wide changes have contributed to improvements in pregnancy outcomes and reduction in maternal mortality [1]. A major contribution to decrease mortality decline can be attributed to more women receiving care from skilled attendants [2]. However, these achievements have not been consistent across countries and regions, with slow pace of change in some localities [3, 4], That could be attributed in part to poor distribution of skilled staff [5] and poor application of clinical skills.

Indonesia's maternal mortality is still very high. In 2012, MMR was reported as 359/100,000 live births [6]. The maternal health programs focused on increasing the number of skilled personnel and promoted facility based births [7]. For example, in East Kalimantan province, where about 90% of women receive antenatal care (ANC) from skilled midwives and more than 84% of women have access to a skilled birth attendant at delivery, the MMR was still high at 175/100,000 [8].

This persistent high maternal mortality despite significant access to the care necessitated a detailed analysis to identify root causes. Considering that maternal deaths occur mainly at the time of labour and delivery and in the postpartum period, the decrease in maternal mortality depends to quite an extent on a well-performing health system [9], and the causes may include ineffective distribution of resources [10] and poor-quality care [11].

We conducted an in-depth analysis of root causes to maternal deaths in district Kutai Kartanegara of East Kalimantan province, to define relevant interventions to improve outcomes. With recommendation and approval by the provincial and district authorities, a team comprising the district health authority, district hospital, and collaborating public health and maternal health academics and researchers set out to identify the root causes of this persistently high MMR.

## 2. Research Setting

Healthcare services in Indonesia are decentralized to the district level, with District's Department of Health responsible for planning, service delivery, and management of primary care services and frontline hospitals. While the frontline secondary care hospitals with basic services are funded and managed by the District Department of Health, the main tertiary specialist hospitals have independent management outside of the District Department of Health, with associated challenges in defining and implementing integrated care between primary care and tertiary care hospital in the district. Mainly the district government provides funding, through its own revenues, for the district services. However, some funds are provided by the central government for subsidized care to poor and as special grants such as for the development of new hospital or extension of services. The provincial department of health provides some financial assistance and technical support for services such as emergency obstetrics care.

The research was conducted for the period from July 2014 to June 2015 in a rural district, Kutai Kartanegara, in East Kalimantan province of about 650,000 people. While topography and access to transport make it difficult for about 100,000 people who live in remote areas to reach hospitals in the city, more than 500,000 people live no more than four hours from the well-equipped district hospital. The district had 32 well-staffed health centres, 179 subhealth centres, 103 doctors, 445 midwives, and 522 nurses. The District Department of Health manages two frontline hospitals placed in the rural areas, about two hours from the capital city of the district. The district's tertiary hospital is located in the capital city and has a well-equipped and well-staffed obstetrics department. Antenatal care, intrapartum care, and postnatal care in primary care are provided by midwives in both public and private sectors in the district. A majority of the women receive ANC, PNC, and labour and delivery care from private midwives in their neighborhoods/villages and from midwives and doctors at the local government healthcare centre.

The district borders with the East Kalimantan province capital city has a tertiary teaching hospital, another large metropolitan city which also has many hospitals and a teaching hospital, and two other districts. Some of the residents of the district who live in the border areas visit these neighboring cities and district for primary care and hospital-based care and for which the district has funding arrangement with the neighboring districts.

In 2013, there were 203,340 women of reproductive age in the district. During that year, out of the 13,688 deliveries in the district, 12,601 were conducted by skilled birth attendants. Despite the high rate of access to ANC and despite 92% being delivered by skilled midwives or at the hospitals, MMR was about 230/100,000 live births. Majority of deaths, 73%, occurred at the hospitals.

## 3. Method

The cases of maternal death in this study were identified through the Indonesian maternal death notification system. Maternal deaths in the hospitals and health centres are reported to the District Department of Health. All deaths of women of reproductive age are investigated by Maternal Health Team at the District Department of Health to confirm if it is a maternal death. Similarly, all deaths in the communities are investigated by the local government health centres with government midwives visiting the family to confirm whether a death is a maternal death. Additionally, the District Department of Health Maternal Health Team receives from the health centres reports of all deaths of woman in reproductive age, investigates, and then conducts verbal autopsies as required by the Indonesian Health System.

For this study, a comprehensive maternal deaths review framework was developed by incorporating safe motherhood concepts*, delays* in decision-making, during transfer and after arriving at the facility [12], barriers to access and sociocultural factors [13], quality of care concepts [14], and signal factors that identify readiness of healthcare facilities [15]. These constructs informed the development of data collection tools, which were used to interview the family members and staff, review the clinical records, and conduct death audits at the hospital.

A questionnaire in the local language was used to conduct verbal autopsy interview with the family members. This detailed structured questionnaire included 104 questions covering information on general health, pregnancy history, use of antenatal and intrapartum care services for the index pregnancy, and a detailed narrative about the terminal event and healthcare received during the terminal event. Additionally, using algorithms information was collected to define if the woman had suffered from preeclampsia, hemorrhage, sepsis, or other complications. The interview with the family took about two hours to complete.

Interviews were conducted with those public and private sector midwives who provided ANC to these 30 women and/or provided care during the terminal events. These interviews were conducted using a questionnaire with questions about general health, risk factors, care provided, and terminal event. The review of deaths at the hospitals included interviews with the staff and review of medical records, including investigations, complications, medication and referrals, timeliness, workloads, and availability of staff, equipment, and products. Information, including ANC received and medication in the Pink Book [pregnant women-held medical record book], was also collected.

Three doctors and three midwives were trained to collect verbal autopsy data. All interviews were conducted by these native language speaking doctors and midwives in Bahasa Indonesia. Six pilot interviews were conducted and minor adjustments to the questionnaires were made. With assistance by the research team, the hospital management constituted a Death Audit Committee. For those who died at the district hospital or another facility in the district, interviews were conducted with the family members, primary care midwife/traditional birth attendant, and the hospital staff. For those who died at home or on the way, interviews were conducted with the family members and primary care providers in addition to reviewing the medical records where available. The district hospital Death Audit Committee included a hospital director, district midwifery services coordinator, an obstetrician, a hospital midwife, and a public health specialist. Medical information contained in the hospital records was collected, and interviews were conducted with the staff.

Interviews were conducted with 30 families. 39 interviews were conducted with ANC providers, as in some cases there were more than one healthcare provider that provided ANC. For those 12 women who died at the district hospital, the hospital Death Audit Committee conducted death audits and the research team reviewed the hospital records in addition to the information provided by ANC providers and family members as well as the information available in the Pink Book (women-held record of health and healthcare during the pregnancy). For those who did not access the district hospital services, the information provided by ANC providers, family members, and the Pink books was reviewed to identify the immediate and root causes.

The data, including detailed terminal event narrative, for the 30 deaths was entered into Excel sheets. The researchers had access to detailed medical records as well. The root-cause analysis focused on organizational and management factors, team environment, individual staff knowledge, skills and practices, and patient characteristics [16]. This framework was used to analyze the comprehensive data collected through interviews with ANC providers, families, and hospital staff and from accessing medical records. This data included information on patients' characteristics such as age, education level, insurance for healthcare and availability of transport, availability of protocols and procedures, integrated or fragmented nature of care, timeliness, referral, and facilitation of transfers to hospitals and follow-up and organizational factors such as workloads, roster, availability of senior consultants, and availability of needed equipment and products.

Based on this data, six case studies were developed and shared with the staff for their observations about what could have been the reasons to ineffective management. A clinically trained health system specialist researcher, two obstetricians, health services managers, and another researcher reviewed the data to identify the contributory factors as defined by Farquhar et al. [17] and adapted by Madzimbamuto et al. [18]. The review considered root causes by reviewing the data to note earliest significant risk or event, how that risk was managed, were any failures in managing those events and risks during antenatal and/or during labour, delivery, or postnatal period. The researchers then discussed the factors that they had identified and developed a consensus about the factors and the underlying explanations. This factors identification and discussion about explanations was also used to categorize deaths as direct or indirect.

## 4. Results

During the 12-month research period, 2014-2015, there were 30 deaths and 14,952 births in the district, with MMR of 200/100,000.

For the 12 deaths that occurred at the government hospitals within the district detailed medical notes were available. For the 10 women who died at the government hospitals outside of the district or at a private hospital, medical records were not available.

Table 1 shows maternal characteristics including age, social context, and factors potentially affecting access to care. Overall, 25 women received care from skilled midwives or obstetricians. Eight of these 25 had ANC at hospitals as well. Only one woman had ANC exclusively by TBA. One woman did not have any ANC by any healthcare provider. Twenty-nine of 30 women died when they were seven months or more through their pregnancy.

Four maternal deaths were during pregnancy and five during labour and 21 women died in the postnatal period, of which 11 died within one day of the delivery, seven deaths occurred within 4–10 days after delivery, and three died more than ten days after the delivery. Fifteen women were delivered by Caesarian Sections (CS).

For the 20 direct maternal deaths, eclampsia/preeclampsia was the immediate cause of death for 45% of the deaths. The other causes included hemorrhage, pulmonary embolism CS complications obstructed labour, and complications of anesthesia (Table 2).

Many women suffered from at least one risk factor. Three women had CS in previous delivery. Four out of 30 were reported to have hypertension prior to the pregnancy or hypercholesterolemia. One woman was pregnant with twin pregnancy. Other risks were grand multiparity in seven women, advanced maternal age in eight women past or suspected tuberculosis in three women, and malaria in one woman (Table 3).

Contributing factors were noted in each of the 30 deaths.  Table 4 provides an example of root-cause analysis questions and results pointing to various organizational, personnel, and other contributory factor that caused death of that woman.  Table 5 shows the contributing factors which played a role in the 30 deaths.

In all 30 deaths personnel factor played a role. The analysis revealed that inadequate knowledge and skills of staff, both in primary care and in hospitals, and failure to follow best practice were the major factors contributing to these deaths. Inadequate knowledge and skills and failure to follow best practices were evident in all three indirect deaths and played a role in 75% of direct deaths as well. The organizational and management factors were noted for 28 (93%). Delay in provision of care, both due to organizational factors such as poor transfer practices from one to the other facility and due to staff failing to recognize the need for urgency, is another major contributing factor. Barriers at family/personal level in 16 (53%) deaths and the distance as a contributing factor also played a role towards 14 deaths (46%). The health services environment and technology factors played a role in 8 of 30 deaths, with unavailability of or delay in procuring blood or blood products in time contributing to 7 of these 8 deaths.

## 5. Discussion

The root-cause analysis in this study provided a comprehensive understanding about how organizational, provider, patient, and community factors affected quality of care. Our study that combined many data collection tools including verbal autopsy and audits, which is an effective measure to identify quality of care factors [19], highlighted not only the factors that contributed to the 30 maternal death in Kutai Kartanegara District but also the details of how each of those factors played a role. The organizational and the personnel factors which played a role as a root cause of almost all the maternal deaths in this study stress further the point that maternal mortality reduction depends on a well-performing health system [9].

Ineffective communication by staff led to poor understanding about which complications could be managed at which of the facilities and when to seek care. The deaths occurring at homes and on the way to a health facility most were due to preeclampsia and hemorrhage, long known causes of MM, for which health providers at first level could be trained and should have been able to assess warning signs and educate women and families about the associated dangers. A major cause of death was eclampsia; hence, midwives, health centre staff, and hospital staff needs to be retrained using protocols about assessment and management of hypertension, facilitating effective referral, defining a delivery plans and follow-ups at home during pregnancy and postnatal periods.

Majority of the women engaged with the formal healthcare services adequately. However, the poor quality of care received by these women is a major concern. In Kutai Kartanegara during the year this research was conducted, 86% deliveries were conducted by skilled birth attendants and 80% women received ANC from skilled healthcare providers (midwives, doctors, and obstetricians) at least 4 times during the pregnancy. Another study that used verbal autopsies to determine factors contributing to maternal mortality reported women having adequate access to care but with quality of care as a major concern [20]. It has been noted that many women in moderate and high maternal mortality areas receive poor-quality care [21, 22]. The reasons to substandard care highlighted by this root-cause analysis include primarily inadequate skills and poor application of protocols and third levels of delays in providing emergency assessment of severity of the condition and delayed referral and procedures. Such substandard care has been documented in other regions [18]. It is documented that at times staff may not be able to effectively assess the severity, in turn causing delays in receiving appropriate care [23]. This was further highlighted in our study as in two-thirds of cases the staff failed to recognize the seriousness of conditions.

In our research setting, the causes of poor quality of healthcare reside in poor organizational capacity and healthcare provider knowledge and practices. Lack of equipment and supplies, such as blood and blood products, deficiency of vital laboratory investigations, and shortage of specialist staff, are major contributory factors. The hospital staff and the Death Audit Committee's observations highlighted the need for improved timeliness and effective care by addressing the shortage of specialists, particularly anesthetists. Similarly, the reviews by hospital staff alerted to the immediate need for timely and improved availability of appropriately crossed matched blood and blood products. Another recommendation based on the Death Audit Committee and hospital staff's observations is to promote a team approach involving multiple specialists for managing complex cases through coordinated assessment and interventions.

Although the data generated by this research does not directly inform about ineffective use of the time of specialists, discussions with the hospital staff and management alerted to unnecessary referrals to specialists and that there is a subtle pressure on specialists to generate resources by providing care to many patients. There is a need to review hospital administration policies and practices that could potentially lead to ineffective use of human resources. A shortage of specialists to be available onsite, particularly during the night shifts, was a major concern. In one case, the unavailability of anesthetist led to less qualified anesthetist nurse to provide anesthesia, the quality of which was questionable and contributed to death. In at least three cases the condition of women deteriorated during the night and the specialist intervention and procedures could only be arranged by the next morning. Another organizational factor was inadequate triage facilities. Poor decision-making by specialists occurs as they may not be able to provide sufficient time and attention to assess risk. The research has identified association between hospital physician workload and length of stay, as an indicator of quality of care [24]. Another factor that compromised the clinical judgment is inadequate capacity to manage preterm babies. At times error in clinical judgment is introduced, such as waiting too long before inducing labour or operating with the intention to avoid having too premature babies which the hospital may not be able to save. Lack of “forward planning” contributed to suboptimal care and delays in procedures. For example, hypertension was managed in hospital with lack of forward plan in terms of target setting for an intended level of blood pressure, planning for change in drugs and/or induction and/or scheduling of CS- if the blood pressure was not lowered to the desired level within the planned timeframe. Another major factor was the [timely] availability of blood and blood products. Hospitals do not have blood banks, and blood and blood products are collected, managed, and supplied by the Red Cross facilities that are often based outside of the hospital premises. Yet another organizational factor is that some of the residents of the district who live in the border areas visit the hospitals in neighboring districts. For women receiving services at the hospitals in the neighboring districts, the district has arrangements to pay fee for service to the hospitals in the neighboring districts. The district, however, has no management or supervisory control and has no arrangement to acquire information about the quality of care in those hospitals. This flow of patient also poses challenges in terms of weaker referral links and poorly coordinated care between the services.

The factors undermining quality of healthcare are present in primary care as well. The inadequate quality of care and inconsistent approach to manage the risks such as preeclampsia in primary care have been noted previously as a major factor in maternal mortality [25]. These factors undermining quality of primary care were obvious in this study as well. The factors affecting quality in primary care included poor personnel skills; for example, vaginal examination being done during active bleeding in a woman who had placenta previa, poor communication between primary care and hospital, primary care centre staff not having information about women receiving care in the private sector, and almost no feedback from the hospitals to primary care settings. Women-held record of pregnancy conditions, assessment, and medication is strongly emphasized by the Indonesian Department of Health. However, it was not utilized effectively; for example, the providers at the hospitals did not use it to communicate back to the primary care provider on how to provide care after discharge. Another reason to poor quality in primary care is the lack of skills in assessing the risks. Management of hypertension was particularly a major area of concern. For example, for six women, hypertension was not effectively managed in the primary care and women were not well informed about the risk. Gradually increasing BP was not picked up by comparing the current with the past BP values or where a 30 mmhg from 90/60 to 110/90 was not seen as an alert. With little information received the women tend to visit only when the medical condition has progressed to a critical stage. Indonesian National and District Governments emphasize correctly and importantly facilitating access to ANC and deliveries by skilled attendants by subsidizing care for poor. A larger majority of women now receive ANC from midwives and at health centres and hospitals. In Kukar, 24 of these 30 women had access to health insurance by the government or employers for highly subsidized care. To further facilitate coverage a National Universal Health Coverage program was introduced in 2014 [26]. A continuous assessment of the impact of increase in utilization on quality of care is needed to define and introduce measures to maintain and improve quality.

Another major contributing factor to the delays in receiving care at the optimum time is the strict hierarchy of referral from the health centre to the district hospital, where some time operative intervention is not feasible, then again to a tertiary hospital. Precious valuable time is usually wasted which results in delays in receiving treatment and leaves the mother in a condition where her life in unsalvageable especially with the limited resources of the tertiary health setting in Indonesia.

It is important to note that social determinants, including factors such as social exclusion, gender equity, education, and employment play a major role towards health [27]. Reduction in poverty and income inequalities and women's political and socioeconomic participation are keys to better health outcomes for women and children [28]. One of the mechanisms by which economic, gender, and social factors may cause poor health outcomes is by compromising access to the needed and appropriate care [29]. Our study focused on assessing the factors causing poor outcomes and deaths despite improved coverage of healthcare services, highlighting the urgent need of improving quality and addressing organizational factors. In Kukar families, 24 of the 30 women had access to personal transport such as motorbikes or small boats, and 29 had attended primary or secondary school. The women were educated enough to know they need facility based care but the substandard care was a problem.

Millennium Development Goal 2015 MMR target was 102 per 100.000 live births. The national average MMR is reported from under 200 [30] to above 300 per 100,000 live births [31]. High MMR in regions such as the Kukar district contribute to this challenge of persistently high maternal mortality. For situations where MMR ranges between 70 and 240, recommendations focus on improving management of complications, timely referral, and improved application of clinical practice guidelines [32]. Recommendations based on this research in Kutai Kartanegara district are summarized in Table 6.

## Figures and Tables

**Table 1:** Maternal characteristics of women who died.

Characteristics	Deaths in hospital (*n* = 22)	Deaths in community (*n* = 8)	Total *n* = 30 (%)
*Age*			
16–22 years	4	3	7 (23.3)
23–29 years	5	1	6 (20.0)
30–35 years	7	2	9 (30.0)
>35 years	6	2	8 (26.7)
*Education*			
None	0	1	1 (3,3)
Primary	12	4	16 (53)
Secondary	4	1	5 (16.6)
>Secondary	6	2	8 (26.6)
*Working status*			
Housewife	20	6	26 (86)
Working in private/govt.	2	2	4 (13)
*Health Care Insurance*			
None	1	5	6 (20)
District govt. Insurance	17	3	20 (66.6)
Employer/other	4	0	4 (13)
*Distance to Health Center*			
10–20 minutes	9	5	14 (46)
>20–45 minutes	5	2	7 (23)
>45–180 minutes	8	1	9 (30)
*Transport at home*			
None	1	5	6 (20)
Motor-Bike	18	3	21 (70)
Small Boat	0	1	1 (3.3)
Motor-bike & a small boat	2	3	5 (16.6)
*General health*			
Good	12	4	16 (53)
High BP/High cholesterol	3	2	5 (16.6)
Shortness of breath	4	1	5 (16.6)
Past or suspected TB	2	1	3 (10)
Other	1	0	1 (3.3)
*Vaccine TT*			
None	3	3	6 (20)
Received 1-2 TT	15	5	20 (66.6)
Missing	4	0	4 (13)
*Delivery Plan by Family*			
None	3	1	4 (13)
TBA, at home	3	3	6 (20)
Midwife, at home	4	2	6 (20)
Midwife, at clinic	4	1	5 (16.6)
Health center	2	0	2 (6.6)
Hospital	3	0	3 (10)
Missing	3	1	4 (13)

**Table 2:** Immediate causes of deaths.

Characteristics	Total (%)	Deaths in Hospital (*n* = 22)	Deaths in Community (Home, health centre, on-the-way to health centre or hospital) (*n* = 8)
Direct maternal deaths	20 (67)	17	3
APH-PPH	3	3	0
Preeclampsia/eclampsia	9	7	2
Obstructed labour	2	2	0
Pulmonary embolism	2	1	1
Anaesthesia related	2	2	0
C-section complications	2	2	0
Indirect maternal death	3 (10)	2	1
Unspecified	7 (23)	3	4

**Table 3:** Maternal obstetric profile of women who died.

Characteristic	Death in the hospital *N* = 22 (%)	Death in the community *N* = 8 (%)	Total *N* = 30 (%)
*Parity*			
0-1	6	3	9 (30)
2–4	12	3	14 (46)
≥5	1	0	1 (3.3)
Missing	4	2	6 (20)
*Gestational Age (months)*			
3 months	1	0	1 (3.3)
7 months	0	1	1 (3.3)
8 months	4	1	5 (16)
9 months	13	6	19 (63)
Missing	4	0	4 (13)
*Antenatal care visits (index pregnancy)*			
No visit	1	2	3 (10)
One visit	1	0	1 (3.3)
Two visits	1	2	3 (10)
Three visits	1	2	3 (10)
Four and above	16	2	18 (59.3)
Missing	2	0	2 (6.6)
*Previous obstetric complications*			
Previous CS	3	0	3 (10)
Previous stillbirth	1	1	2 (7)
Previous abortion	1	1	2 (7)
Previous miscarriage	0	0	0 (0)
Previous postpartum hemorrhage	1	0	1 (3.3)
*First attendant at delivery*			
Traditional birth attendant			7 (23)
Trained midwife			13 (43)
Midwife at the health centre			3 (10)
Government Hospital			2 (7)
Private Hospital			0 (0)
Private midwife clinic			0 (0)
Family member			1 (3.3)
Missing			4 (13)

**Table 4:** Root-cause analysis example: factors contributing to a death due to hemorrhage cause of death: hemorrhagic shock.

Questions & Reasons	How to address the contributing factors
*Why the hemorrhage and shock could not be managed*: Delay in operation due to the absence of senior obstetrician, Delay in blood transfusion, Incorrect assessment of blood loss with inadequate amount of blood transfused. No clotting screening done and no renal function assessed	Improve rosters and policies for timely availability of specialists, development of midwifery risk assessment teams at ER Refresher training for pre-op management and preparation Review policies to plan timely provision of blood, blood products and other essential supplies

*Why hemorrhage occurred*: Posterior wall of the uterus ruptured for obstructed labour due to big baby (4 kg), with delays in accessing care at the hospital	Refresher training for better assessment of risks, management of risks hypertension, diabetes, and effective course of action for complications such as obstructed labour

*Why delays in accessing care at the hospital*: Delay by the midwife at the centre to facilitate referral and transfer to woman to hospital, Poor referral communication causing woman to arrive first at a hospital with no CS facility. Delays at the terminal event occurred as there was no hospital registration and delivery plan	Develop, implement and monitor protocols for follow ups to assess if the referral was taken. Provide primary care workers, particular those midwives who are providing labour and delivery care in private sector, information about what services are available in which of the facilities

*Why there was no effective delivery plan*: Earlier ANC provider midwife failed to effectively advise the woman to deliver in the hospital despite that there was a previous still birth and current baby is big, and despite that the nearest health care facility which can provide Caesarean Section is 6 hour away	Train primary care staff for risk assessment and for communicating the risk. Protocols for early assessment (first trimester) and subsequent categorization into high, intermediate and low risk, with each category having a plan of where to deliver. The plan should be included in the Pink Book

*Why ANC provider failed to effectively advise*: Lack of application of best practice protocol and failure to recognize clinically big baby (4 kg baby at 8 months) and no request for an ultrasound scan, Risks were not carefully assessed	Retrain midwives, with a focus on best practice protocols, referrals, communication skills, assessment and management of risks with case studies based on situation in the district

*Why risks were not fully assessed early for a 37 years old, G5* with history of abortion and stillbirth, and who lived 6 hours from a hospital: Family planning services are un-integrated and are the responsibility of another department, ANC sessions are rushed with inadequate emphasis on geography, access, age, FP, past history and planning and communication for follow up	Primary care services in this district must include family planning, actively supporting woman offering them a selection of methods. Improved health education as part of centre based and home based ANC provision

**Table 5:** Factors contributing to maternal death.

Contributing factors	Total deaths	Immediate cause of maternal death			Unsure if the factor played a role
		Direct	Indirect	Unknown	
	30	20	3	7	

*Organization*	**28 (93%)**				
Poor organization/management (both in primary and tertiary care)	9	7	1	0	5
Lack of policy/protocol/guidelines	13	12	1	0	2
Inadequate staff	4	4	0	0	1
Inadequate access to senior clinical staff	13	9	2	2	2
Failure/delay in emergency response	15	11	1	3	2
Delay in procedures	11	8	0	3	4
Poor system/process for sharing information (between primary and tertiary care)	7	4	1	3	4
Delay in Access to Test Results	2	1	1	0	4

*Personnel*	**30 (100%)**				
Knowledge and skills lacking	24	15	3	6	4
Delay in emergency response	14	9	1	4	2
Poor communication	9	6	2	1	3
Failure to seek supervision/help	13	7	2	4	3
Failure to follow best practice (hospital for 13 women, primary care for 12)	25	18	3	4	3
Lack of recognition of seriousness	20	13	2	5	4

*Equipment & supplies*	**7 (23%)**				
Malfunction/failure	1	1	0	0	1
Supplies (blood, FFP, drugs, etc.) out of stock, unavailable on premises	7	6	0	1	0

*Environment*	**14 (47%)**				
Geography as contributory factor	14	9	1	4	1

*Barriers at personal/family*	**16 (53%)**				
Lack of recognition of seriousness	16	9	2	5	1
Not Eligible for free care/financial difficulty	5	3	1	1	0

Adapted from Farquhar et al. 2011 and Madzimbamuto et al. 2014.

**Table 6:** Recommendations to address the contributing factors.

Contributing factors	Recommendations
Failure to follow best practice protocols	District Health Department should institute a system of closer supervision & support, with maternal health team having sufficient number of midwife-supervisors working closely with the health centre and private midwives

Inability to manage deterioration	In hospitals, provide CME, and where possible place trainers on-site, retrain staff using standard courses particularly for preeclampsia and postpartum hemorrhage and effective use of early warning system. In primary care retrain staff for improved skills particularly for BP measurement and management

Poorly resourced facilities	Strengthen midwifery at the sub-district hospitals. Review staffing needs and retrain staff at the centres particularly those that are two or more hours from the district hospital

Missing essential service, such as blood products	Support the hospital management for decision to initiate or relocate services on premises

Ineffective communication	Conduct focused training for midwifery supervisors and heads of primary care centres, to communicate and develop delivery plans with the women, document sufficient details of the condition, and effectively use hotline with calls to hospitals before and during transfer of women

Unintegrated care and poor referrals	Develop protocols for early assessment and subsequent categorization into high, intermediate and low risk, with each category having a clear plan of where to deliver. Train staff using these protocols including information about capability of each of the district hospitals in terms of what services are available. Additionally, there is a need to train staff and emphasize on assessment of risks posed by concomitant illnesses with reference to the locally prevalent diseases such as TB, malaria and dengue, and provision of comprehensive and integrated care through a team approach.

Ineffective family planning services	Reemphasize a strong focus on family planning as part of maternal health care services in both primary care and at the hospital. Retrain staff to provide care to women with unmet need and potential unplanned pregnancies with a particular focus on multipara and age beyond 30

Many women suffering from hypertensive conditions	Investigate eating & nutritional practices (e.g. salt intake)

## Additional Points

*Limitation*. Hospital-based audits could only be conducted for those deaths that occurred at the district hospital. The research team did not have access to records from hospitals in other districts. Similarly, the research team did not have the mandate to review blood bank services outside of the hospital. Such “outside of health facilities factor” requires further research.
